# Influencing factors of length of stay among repeatedly hospitalized patients with mood disorders: a longitudinal study in China

**DOI:** 10.1186/s12991-024-00497-y

**Published:** 2024-04-25

**Authors:** Feng Xu, Peixia Cheng, Jiaying Xu, Xiaonan Wang, Zhen Jiang, Huiping Zhu, Hua Fan, Qian Wang, Qi Gao

**Affiliations:** 1 Department of Epidemiology and Health Statistics, School of Public Health, Capital Medical University, 10 Xitoutiao, Youanmen Wai, Beijing, 100069 China; 2 Capital Medical University Affiliated Beijing Anding Hospital, Beijing, China

**Keywords:** Length of stay, Mood disorders, Repeatedly hospitalized patients, Influencing factors

## Abstract

**Background:**

Patients with mood disorders usually require repeated and prolonged hospitalization, resulting in a heavy burden on healthcare resources. This study aims to identify variables associated with length of stay(LOS) of repeatedly hospitalized patients with mood disorders and to provide information for optimizing psychiatry management and healthcare resource allocation.

**Methods:**

Electronic medical records (EMRs) of repeatedly hospitalized patients with mood disorders from January 2010 to December 2018 were collected and retrospectively analyzed. Chi-square and *t-*test were adopted to investigate the differences in characteristics between the two groups of short LOS and long LOS. Generalized estimating equation (GEE) was conducted to investigate potential factors influencing LOS.

**Results:**

A total of 2,009 repeatedly hospitalized patients with mood disorders were enrolled, of which 797 (39.7%) had a long LOS and 1,212 (60.3%) had a short LOS. Adverse effects of treatment, continuous clinical manifestation, chronic onset type, suicide attempt, comorbidity and use of antidepressants were positively associated with long LOS among all repeatedly hospitalized patients with mood disorders (*P* < 0.050). For patients with depression, factors associated with long LOS consisted of age, monthly income, adverse effects of treatment, continuous clinical manifestation, suicide attempt and comorbidity (*P* < 0.050). Whereas, for patients with bipolar disorder (BD), adverse effects of treatment, four or more hospitalizations and use of antidepressants contributed to the long LOS (*P* < 0.050). Influencing factors of LOS also vary among patients with different effectiveness of treatment.

**Conclusion:**

The LOS in repeatedly hospitalized patients with mood disorders was influenced by multiple factors. There were discrepancies in the factors affecting LOS in patients with different diagnoses and effectiveness of treatment, and specific factors should be addressed when evaluating the LOS.

## Background

Mood disorders are the most prevalent category of mental disorders that can simultaneously affect one’s emotions, energy, and motivation [1], and are usually categorized into two types: depression and bipolar disorder (BD). In 2019, approximately 970 million individuals worldwide were affected by mental illness [2], the number of people with depression was exceeding 280 million and 40 million people experienced BD [3]. In China, mood disorders have emerged as the second most prominent lifetime disorders, with weighted lifetime prevalence rates of depression and BD as high as 6.8% and 0.6% in 2013–2014, respectively [4], and the growth in prevalence rates has not been alleviated [5]. Mood disorders have resulted in a tremendous burden on the quality of life of patients and their caregivers, as well as on the medical, psycho-social, and economic development of society.

Mood disorders tend to be chronic and recurrent, with vast majority of patients requiring repeated hospitalization. As the prevalence of mental disorders continues to rise in China, healthcare resources available to patients are already in shortage [6]. And it is particularly imperative to allocate healthcare resources more appropriately to ensure that those most in need receive timely and prioritized treatment. Reducing the length of stay (LOS) for patients with mental disorders [7] is one of the most appropriate ways to optimize the rational allocation of healthcare resources.

Previous researches have documented that the average LOS of patients with mood disorders varied dramatically between countries, but the LOS for patients with mood disorders in almost all countries was long, with a minimum of up to about 20 days. The average LOS in acute mental health services for patients with depression and BD in the UK were 22.88 days and 53.88 days, respectively [8]; for patients with mood disorders in Switzerland the average LOS was 37.2 days [9]; and for patients with BD in Italy it was 30.45 days [10]. In China, data on LOS of patients with mood disorders in studies from different hospitals were also inconsistent, with data from Beijing Anding Hospital indicating an average LOS of 28 days for patients with BD [11], and data from the Second Xiangya Hospital of Central South University indicated an average LOS of 17 days for patients with depression [12]. Prolonged LOS not only negatively impacts patients’ confidence in treatment and employment opportunities, but also leads to increased expenditure and inefficient allocation of healthcare resources.

Previous studies have documented that factors such as sociodemographic characteristics (e.g., gender, age and marital status) [13], comorbidities [14], and history of hospitalization [15] may influence the LOS for patients with mental disorders. However, prior studies have only analyzed data at a single point in time [8, 16, 17], and investigations on longitudinal data for patients with mood disorders who have repeatedly hospitalized or multiple treated remains limited. Consequently, by utilizing longitudinal electronic medical record data from Beijing Anding Hospital, this study analyzed the variables associated with LOS of repeatedly hospitalized patients with mood disorders. Beijing Anding Hospital, situated in the capital of China, is recognized as one of the top five psychiatric hospitals in China. With a bed capacity of 800, the hospital has been catering to patients from various regions of China. In 2010, the hospital implemented an electronic medical record (EMR) system, which underwent thorough debugging to ensure data stability. The aim of this study is to provide valuable information that can be used to optimize psychiatric management and healthcare resource allocation.

## Methods

### Data source

Our study population consists of patients with mood disorders who were repeatedly hospitalized at Beijing Anding Hospital two or more times from 1 January 2010 to 31 December 2018. Patients were enrolled according to the following eligibility criteria: (1) patients diagnosed with mood disorders at the time of their first hospitalization according to the International Classification of Diseases-10th Revision (ICD-10); (2) aged 18 years or older ;(3) two or more documented hospitalizations from 1 January 2010 to 31 December 2018. Patients were excluded if they were not Chinese nationals or if their EMRs were incomplete.

Initially, a total of 5,253 potentially eligible patients with mood disorders who had been repeatedly hospitalized were retained. After applying the inclusion and exclusion criteria, a final sample size of 2,009 individuals was included in this study. Patients’ information such as sociodemographic characteristics, date of hospitalization and discharge, clinical diagnosis at the time of discharge, patient’s chief complaint, history of present illness and past medical history were extracted from the EMRs using text mining.

The discharge criteria for patients with mood disorders are as follows (based on the assessment of deputy chief physician or above): (1) The risks or behaviors of suicide, self-harm, or harm to others had been alleviated, which patients exhibiting upon admission. (2) Disease symptoms should have significantly improved, meanwhile, the medication dosage (if the patient underwent medication treatment) should be stabilized and no significant adverse drug reactions.

This study was approved by the Institutional Review Board of Capital Medical University (Z2019SY006).

### Variables

The study focused on the LOS of patients in the hospital, which is defined as the time from admission to discharge. LOS was calculated by subtracting the admission date from the discharge date. Referring to previous similar study [10], we used the mean LOS as the cut-off value, and categorized the LOS of patients at each time admission into “long LOS” (≥ 32 days) and “short LOS” (< 32 days).

The independent variables included sociodemographic characteristics, medical history, disease-related characteristics and treatment-related characteristics. Sociodemographic characteristics encompassed variables such as gender, age, occupation, monthly income, place of residence, ethnicity, marital status, only child, interpersonal relationship and having children. “Age” was reported in years of the individual at the time of hospital admission. “Place of residence” referred to the patients’ usual living location, which is divided into Beijing and non-Beijing. “Ethnicity” was classified as Han and minority, with the minority category including 55 different ethnic minorities (e.g., Zhuang, Man, Hui, Miao, Uyghur, Tujia, Yi, Mongo etc.). “Marital status” was grouped into single, married and divorced/widowed. “Only child” referred to whether the patients with mood disorders was a single child. “Interpersonal relationship” was categorized by good, general and bad. The data was derived from patients’ self-reports through the question “Do you feel that you have harmonious interpersonal relationships?”. If the patient was unable to provide information, a family member or caregiver would answer on their behalf. “Having children” referred to whether the patients have children.

Medical history referred to history of suicide, history of various diseases (e.g., respiratory diseases, cardiovascular diseases, genitourinary diseases, digestive disease, endocrine disorders, neurological diseases, infectious disease and trauma), drug and food allergies, suicide, vaccinations, smoking and drinking. “Vaccination” is the variable used to obtain information on the patient’s previous vaccinations, defined as “yes” if the patient has received any of the vaccines in the National Immunization Program (NIP).

Disease-related characteristics consisted of self-care ability, precipitating factor of diseases, adverse effects of treatment, clinical manifestation, onset type, suicide behavior at admission, hospitalization frequency, duration of disease and comorbidity. “Self-care ability” was grouped into unable, partial and able. “Precipitating factor of diseases” referred to whether there are specific factors or events that lead to the onset of mood disorder in a patient. “Adverse effects of treatment” was extracted as a dichotomous variable categorized as yes and no according to whether the patient experienced an adverse reaction during treatment. “Clinical manifestation” was the characteristics of the disease since the patient’s first episode and divided into two categories: intermittent and continuous. “Onset type” was grouped into acute, sub-acute and chronic. “Suicide behavior” was divided into no, suicide ideation and suicide attempt. The professionals asked the patients or their guardians about the suicidal behaviors. “Hospitalization frequency” was referred to the number of times a patient had been admitted to Beijing Anding Hospital. It grouped into three categories: 2, 3 and ≥ 4. “Duration of disease” referred to the time experienced by the patient since the first onset of the disease. “Comorbidity” referred to the patients with mood disorders suffered from physical medical problems. Our study categorized comorbidity as yes and no based on the discharge diagnosis.

Treatment-related characteristics included modified electroconvulsive therapy (MECT), treatment effectiveness, whether use psychotropic drugs. MECT is a psychiatric treatment where a generalized seizure (without muscular convulsions) is electrically induced to manage mental disorders. “Treatment effectiveness” was grouped into cure, improvement and not healed. The use of psychotropic drugs included whether use antipsychotics, whether use moodstabilizers, whether use antidepressants, whether use anxiolytics.

### Statistical analysis

Chi-square and *t-*test were adopted to investigate the distribution of characteristics among patients with different disease types at initial hospitalization and the differences in characteristics between the two groups of short LOS and long LOS . Generalized estimating equation (GEE), a statistical model developed on the basis of generalized linear models designed to deal with longitudinal data, was performed to explore the influencing factors of LOS among repeatedly hospitalized patients with BD and depression. The GEE model can effectively utilize each measurement values from the same patients and take into account the correlation between these measurements to void unnecessary loss of information. Especially considering that patients may have varying intervals and number of hospitalizations, the GEE model is a suitable method for data analysis. In our study, the GEE model employed a binary logistic model with a logit link and an exchangeable working correlation matrix.

The results of GEE model were demonstrated using forest plots. All analyses and plots were conducted using SPSS 26.0 (SPSS Institute Inc., USA) and R 4.1.3. Two-tailed *p* < 0.050 was considered statistically significant.

## Results

### Distribution of characteristics among study population at first hospitalization

Table 1 presents the distribution of characteristics according to the diagnosis. A total of 2,009 repeatedly hospitalized patients were enrolled in this study, of which 1,179 (58.7%) and 830 (41.3%) were diagnosed with BD and depression, respectively, at the time of initial hospitalization. The mean age of the study population was 37.41 ± 15.25 years, with the majority of patients being female (62.4%), residing in Beijing (60.0%), Han Chinese (95.9%), and married (54.6%). 71.2% of the study participants were hospitalized twice, 19.0% were hospitalized three times, and 9.8% were hospitalized four or more times. The mean duration of all patients was 5.26 years, and 4.87 years, 5.80 years in patients with BD and depression, respectively. 1174 (58.4%) patients accepted the treatment of MECT, of which 63.9% was BD patients.

**Table 1:** Distribution of characteristics among patients with different disease types at initial hospitalization

Variable		All		BD		Depression		*χ*^*2*^ or *t*		*P*
**Overall**		2009		1179(58.7%)		830(41.3%)				
**Gender, n (%)**								16.426		**< 0.001**
Male		756(37.6)		487(64.4)		269(35.6)				
Female		1253(62.4)		692(55.2)		561(44.8)				
**Age, (years) mean ± SD**		37.41 ± 15.25		32.06 ± 12.36		45.01 ± 15.73		-19.806		**< 0.001**
**Occupation, n (%)**								105.021		**< 0.001**
Worker/farmer		62(3.1)		37(59.7)		25(40.3)				
Employee		223(11.1)		130(58.3)		93(41.7)				
Freelancer		1142(56.8)		638(55.9)		504(44.1)				
Soldier or student		155(7.7)		127(81.9)		28(18.1))				
Retiree		130(6.5)		37(28.5)		93(71.5)				
Unemployed		297(14.8)		210(70.7)		87(29.3)				
**Monthly income, (yuan) n (%)**								8.966		**0.030**
< 1000		234(11.6)		154(65.8)		80(34.2)				
1000–3000		927(46.1)		553(59.7)		374(40.3)				
3000–5000		467(23.2)		265(56.7)		202(43.3)				
≥ 5000		381(19.1)								
**Residence, n (%)**								0.884		0.347
Beijing		1205(60.0)		697(57.8)		508(42.2)				
Non-Beijing		804(40.0)		482(60.0)		322(40.0)				
**Ethnicity, n (%)**								0.434		0.510
Han		1927(95.9)		1128(58.5)		799(41.5)				
Minority		82(4.1)		51(62.2)		31(37.8)				
**Marital status, n (%)**								191.153		**< 0.001**
Single		753(37.5)		589(78.2)		164(21.8)				
Married		1097(54.6)		508(46.3)		589(53.7)				
Divorced or widowed		159(7.9)		82(51.6)		77(48.4)				
**Only child, n (%)**								70.599		**< 0.001**
No		1436(71.5)		759(52.9)		677(47.1)				
Yes		573(28.5)		420(73.3)		153(26.7)				
**Interpersonal relationship, n (%)**								23.681		**< 0.001**
Bad		160(8.0)		112(70.0)		48(30.0)				
General		823(41.0)		515(62.6)		308(37.4)				
Good		1026(51.1)		552(53.8)		474(46.2)				
**Having children, n (%)**								193.951		**< 0.001**
No		862(42.9)		658(76.3)		204(23.7)				
Yes		1147(57.1)		521(45.4)		626(54.6)				
**Suicide history, n (%)**								2.146		0.143
No		1567(78.0)		933(59.5)		634(40.5)				
Yes		442(22.0)		246(55.7)		196(44.3)				
**History of respiratory diseases, n (%)**								0.625		0.429
No		1879(93.5)		1107(58.9)		772(41.1)				
Yes		130(6.5)		72(55.4)		58(44.6)				
**History of cardiovascular diseases, n (%)**								13.254		**< 0.001**
No		1697(84.5)		1081(63.7)		616(36.3)				
Yes		312(15.5)		98(31.4)		214(68.6)				
**History of genitourinary diseases, n (%)**								13.254		**< 0.001**
No		1855(92.3)		1110(59.8)		745(40.2)				
Yes		154(7.7)		69(44.8)		85(55.2)				
**History of digestive diseases, n (%)**								19.440		**< 0.001**
No		1762(87.7)		1066(60.5)		696(39.5)				
Yes		247(12.3)		113(45.7)		134(54.3)				
**History of endocrine disorders, n (%)**								28.456		**< 0.001**
No		1835(91.3)		1110(60.5)		725(39.5)				
Yes		174(8.7)		69(39.7)		105(60.3)				
**History of neurological diseases, n (%)**								21.913		**< 0.001**
No		1895(94.3)		1136(59.9)		759(40.1)				
Yes		114(5.7)		43(37.7)		71(62.3)				
**History of drug allergies, n (%)**								1.444		0.229
No		1808(90.0)		1069(59.1)		739(40.9)				
Yes		201(10.0)		110(54.7)		91(45.3)				
**History of food allergies, n (%)**								6.487		**0.011**
No		1927(95.9)		1142(59.3)		785(40.7)				
Yes		82(4.1)		37(45.1)		45(54.9)				
**Vaccinations, n (%)**								7.005		**0.008**
No		158(7.9)		77(48.7)		81(51.3)				
Yes		1851(92.1)		1102(59.5)		749(40.5)				
**History of infectious diseases, n (%)**								0.143		0.705
No		1935(96.3)		1134(58.6)		801(41.4)				
Yes		74(3.7)		45(60.8)		29(39.2)				
**History of major trauma, n (%)**								0.384		0.535
No		1793(89.2)		1048(58.4)		745(41.6)				
Yes		216(10.8)		131(60.6)		85(39.4)				
**Smoking, n (%)**								22.559		**< 0.001**
No		1549(77.1)		865(55.8)		684(44.2)				
Yes		460(22.9)		314(68.3)		146(31.7)				
**Drinking, n (%)**								6.322		**0.012**
No		1681(83.7)		966(57.5)		715(42.5)				
Yes		328(16.3)		213(64.9)		115(35.1)				
**Self-care ability, n (%)**								8.826		**0.012**
Unable		15(0.7)		6(40.0)		9(60.0)				
Partial		155(7.7)		76(49.0)		79(51.0)				
Able		1839(91.5)		1097(59.7)		742(40.3)				
**Precipitating factor, n (%)**								19.935		**< 0.001**
No		1232(61.3)		771(62.6)		461(37.4)				
Yes		777(38.7)		408(52.5)		369(47.5)				
**Adverse effects of treatment, n (%)**								7.006		**0.008**
No		1300(64.7)		735(56.5)		565(43.5)				
Yes		709(35.3)		444(62.6)		265(37.4)				
**Clinical manifestation, n (%)**								33.240		**< 0.001**
Intermittent		1350(67.2)		852(63.1)		498(36.9)				
Continuous		659(32.8)		327(49.6)		332(50.4)				
**Onset type, n (%)**								16.506		**< 0.001**
Acute		464(23.1)		310(66.8)		154(33.2)				
Sub-acute		349(17.4)		194(55.6)		155(44.4)				
Chronic		1196(59.50		675(56.4)		521(43.6)				
**Suicide behavior, n (%)**								165.804		**< 0.001**
No		1255(62.5)		874(69.6)		381(30.4)				
Suicide ideation		526(26.2)		216(41.1)		310(58.9)				
Suicide attempt		228(11.3)		89(39.0)		139(61.0)				
**Hospitalization frequency, n (%)**								2.470		0.291
2		1430(71.2)		824(57.6)		606(42.4)				
3		382(19.0)		232(60.7)		150(39.3)				
≥ 4		197(9.8)		123(62.4)		74(37.6)				
**Duration, (years) Mean ± SD**		5.26 ± 7.28		4.87 ± 6.63		5.80 ± 8.10		-2.728		**0.006**
**MECT, n(%)**								31.481		**< 0.001**
No		835(41.6)		429(51.4)		406(48.6)				
Yes		1174(58.4)		750(63.9)		328(36.1)				
**Comorbidity, n(%)**								30.443		**< 0.001**
No		471(23.4)		328(69.6)		143(30.4)				
Yes		1538(76.6)		851(55.3)		687(44.7)				
**Treatment effectiveness, n(%)**								4.623		0.099
Cure		276(13.7)		178(64.5)		98(35.5)				
Improvement		1653(82.3)		953(57.7)		700(42.3)				
Not healed		80(4.0)		48(60.0)		32(40.0)				
**Antipsychotics, n(%)**								305.769		**< 0.001**
No		378(18.8)		71(18.8)		307(81.2)				
Yes		1631(81.2)		1108(67.9)		523(32.1)				
**Moodstabilizers, n(%)**								1245.390		**< 0.001**
No		745(37.1)		61(8.2)		684(91.8)				
Yes		1264(62.9)		1118(88.4)		146(11.6)				
**Antidepressants, n(%)**								1067.434		**< 0.001**
No		1083(53.9)		995(91.9)		88(8.1)				
Yes		926(46.1)		184(19.9)		742(80.1)				
**Anxiolytics, n(%)**								122.815		**< 0.001**
No		1491(74.2)		982(65.9)		509(34.1)				
Yes		518(25.8)		197(38.0)		321(41.3)				

**Fig. 1:**
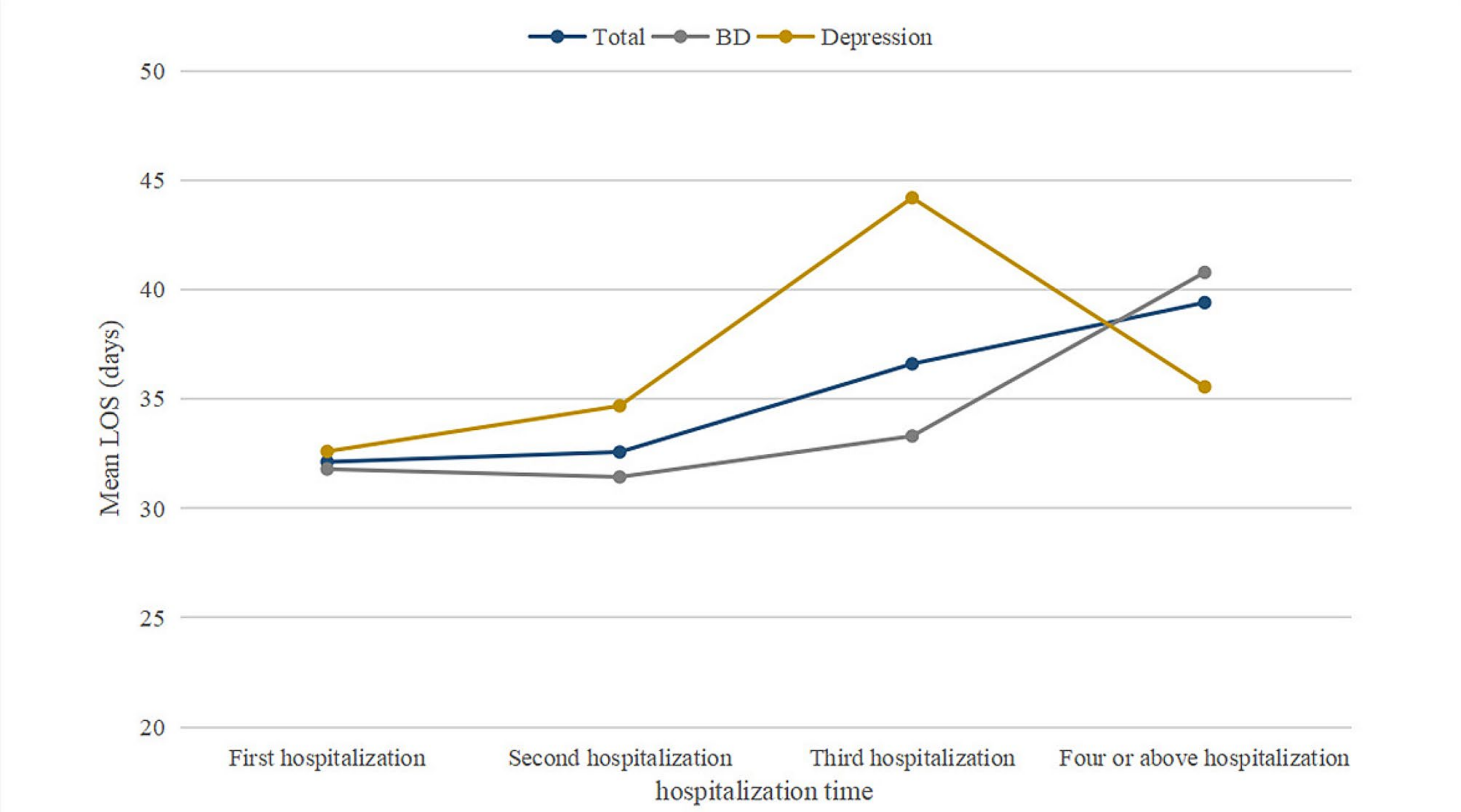
The change in mean LOS among repeatedly hospitalized patients with mood disorders

Mean LOS was significantly higher for mood disorder patients as the number of hospitalizations increased. The mean LOS of patients with mood disorders was 32.12 ± 22.70 days, 32.57 ± 21.98 days, 36.60 ± 33.42days and 39.40 ± 65.06 days at the first, second, third, and fourth or above hospitalizations, respectively. Moreover, the mean LOS of patients with depression was consistently longer than that of patients with BD until the fourth or above hospitalization (Fig. 1).

As shown in Table 2, the comparison of baseline characteristics between the short LOS group and long LOS group of repeatedly hospitalized patients with mood disorders demonstrated statistically significant differences in various factors. These included occupation, monthly income, ethnicity, marital status, interpersonal relationship, whether have children, history of respiratory diseases, history of vaccinations, smoking, drinking, self-care ability, adverse effects of treatment, and precipitating factor of diseases, MECT, treatment effectiveness, whether use antipsychotics, whether use anxiolytics(*P*<0.050).

**Table 2:** The comparison of characteristics at initial hospitalization between two LOS groups

Variable		Short LOS		Long LOS		*χ*^*2*^ or *t*		*P*
**Overall**		1212(60.3%)		797(39.7%)				
**Gender, n (%)**						3.515		0.061
Male		476(63.0)		280(37.0)				
Female		736(58.7)		517(41.3)				
**Age, (years) mean ± SD**		37.68 ± 14.82		37.00 ± 15.87		0.965		0.335
**Occupation, n (%)**						32.770		**< 0.001**
Worker/farmer		35(56.5)		27(43.5)				
Employee		133(59.6)		90(40.4)				
Freelancer		737(64.5)		405(35.5)				
Soldier or student		77(49.7))		78(50.3)				
Retiree		56(43.1)		74(56.9)				
Unemployed		174(58.6)		123(41.4)				
**Monthly income, (yuan) n (%)**						13.122		**0.004**
< 1000		141(60.3)		93(39.7)				
1000–3000		523(56.4)		404(43.6)				
3000–5000		296(63.4)		171(36.6)				
≥ 5000		252(66.1)		129(33.9)				
**Residence, n (%)**						0.549		0.459
Beijing		719(59.7)		486(40.3)				
Non-Beijing		493(61.3)		311(38.7)				
**Ethnicity, n (%)**						5.891		**0.015**
Han		1152(59.8)		775(40.2)				
Minority		60(73.2)		22(26.8)				
**Marital status, n (%)**						14.945		**0.001**
Single		419(55.6)		334(44.4)				
Married		704(64.2)		393(35.8)				
Divorced or widowed		89(56.0)		70(44.0)				
**Only child, n (%)**						0.005		0.945
No		867(60.4)		569(39.6)				
Yes		345(60.2)		228(39.8)				
**Interpersonal relationship, n (%)**						7.047		**0.029**
Bad		96(60.0)		64(40.0)				
General		469(57.0)		354(43.0)				
Good		647(63.1)		379(36.9)				
**Having children, n (%)**						9.263		**0.002**
No		487(56.5)		375(43.5)				
Yes		725(63.2)		422(36.8)				
**Diagnosis, n (%)**						1.511		0.219
BD		698(59.2)		481(40.8)				
Depression		514(61.9)		316(38.1)				
**Suicide history, n (%)**						2.602		0.107
No		960(61.3)		607(38.7)				
Yes		252(57.0)		190(43.0)				
**History of respiratory diseases, n (%)**						5.307		**0.021**
No		1146(61.0)		733(39.0)				
Yes		66(50.8)		64(49.2)				
**History of cardiovascular diseases, n (%)**						0.361		0.548
No		1019(60.0)		678(40.0)				
Yes		193(61.9)		119(38.1)				
**History of genitourinary diseases, n (%)**						0.448		0.503
No		1123(60.5)		732(39.5)				
Yes		89(57.8)		65(42.2)				
**History of digestive diseases, n (%)**						1.231		0.267
No		1055(59.9)		707(40.1)				
Yes		157(63.6)		90(36.4)				
**History of endocrine disorders, n (%)**						1.299		0.254
No		1100(59.9)		735(40.1)				
Yes		112(64.4)		62(35.6)				
**History of neurological diseases, n (%)**						1.296		0.255
No		1149(60.6)		746(39.4)				
Yes		63(55.3)		51(44.7)				
**History of drug allergies, n (%)**						0.002		0.968
No		1091(60.3)		717(39.7)				
Yes		121(60.2)		80(39.8)				
**History of food allergies, n (%)**						3.013		0.083
No		1155(59.9)		772(40.1)				
Yes		57(69.5)		25(30.5)				
**History of vaccinations, n (%)**						10.712		**0.001**
No		76(48.1)		82(51.9)				
Yes		1136(61.4)		715(38.6)				
**History of infectious diseases, n (%)**						2.369		0.124
No		1161(60.0)		774(40.0)				
Yes		51(68.9)		23(31.1)				
**History of major trauma, n (%)**						0.01		0.919
No		1081(60.3)		712(39.7)				
Yes		131(60.6)		85(39.4)				
**Smoking, n (%)**						9.561		**0.002**
No		906(58.5)		643(41.5)				
Yes		306(66.5)		154(33.5)				
**Drinking, n (%)**						10.389		**0.001**
No		988(58.8)		693(41.2)				
Yes		224(68.3)		104(31.7)				
**Self-care ability, n (%)**						10.303		**0.006**
Unable		7(46.7)		8(53.3)				
Partial		76(49.0)		79(51.0)				
Able		1129(61.4)		710(38.6)				
**Precipitating factor n (%)**						7.501		**0.006**
No		714(58.0)		518(42.0)				
Yes		498(64.1)		279(35.9)				
**Adverse effects of treatment, n (%)**						31.412		**< 0.001**
No		843(64.8)		457(35.2)				
Yes		369(52.0)		340(48.0)				
**Clinical manifestation, n (%)**						3.612		0.057
Intermittent		834(61.8)		516(38.2)				
Continuous		378(57.4)		281(42.6)				
**Onset type n (%)**						3.584		0.167
Acute		297(64.0)		167(36.0)				
Sub-acute		210(60.2)		139(39.8)				
Chronic		705(58.9)		491(41.1)				
**Suicide behavior, n (%)**						4.989		0.083
No		760(60.6)		495(39.4)				
Suicide ideation		329(62.5)		197(37.5)				
Suicide attempt		123(53.9)		105(46.1)				
**Hospitalization frequency, n (%)**						3.316		0.191
2		871(60.9)		559(39.1)				
3		234(61.3)		148(38.7)				
≥ 4		107(54.3)		90(45.7)				
**Duration, (years) Mean ± SD**		5.37 ± 7.11		5.08 ± 7.55		0.870		0.384
**MECT, n(%)**						17.539		**< 0.001**
No		549(65.7)		286(34.3)				
Yes		663(56.5)		511(43.5)				
**Comorbidity, n(%)**						1.914		0.166
No		297(63.1)		174(36.9)				
Yes		915(59.5)		623(40.5)				
**Treatment effectiveness, n(%)**						103.117		**< 0.001**
Cure		105(38.0)		171(62.0)				
Improvement		1030(62.3)		623(37.7)				
Not healed		77(96.2)		3(3.8)				
**Antipsychotics, n(%)**						5.981		**0.014**
No		249(65.9)		129(34.1)				
Yes		963(59.0)		668(41.0)				
**Moodstabilizers, n(%)**						3.408		0.065
No		469(63.0)		276(37.0)				
Yes		743(58.8)		521(41.2)				
**Antidepressants, n(%)**						0.058		0.809
No		656(60.6)		427(39.4)				
Yes		556(60.0)		370(40.0)				
**Anxiolytics, n(%)**						11.479		**0.001**
No		867(58.1)		624(41.9)				
Yes		345(66.6)		173(33.4)				

### GEE model of LOS among study population

**Fig. 2:**
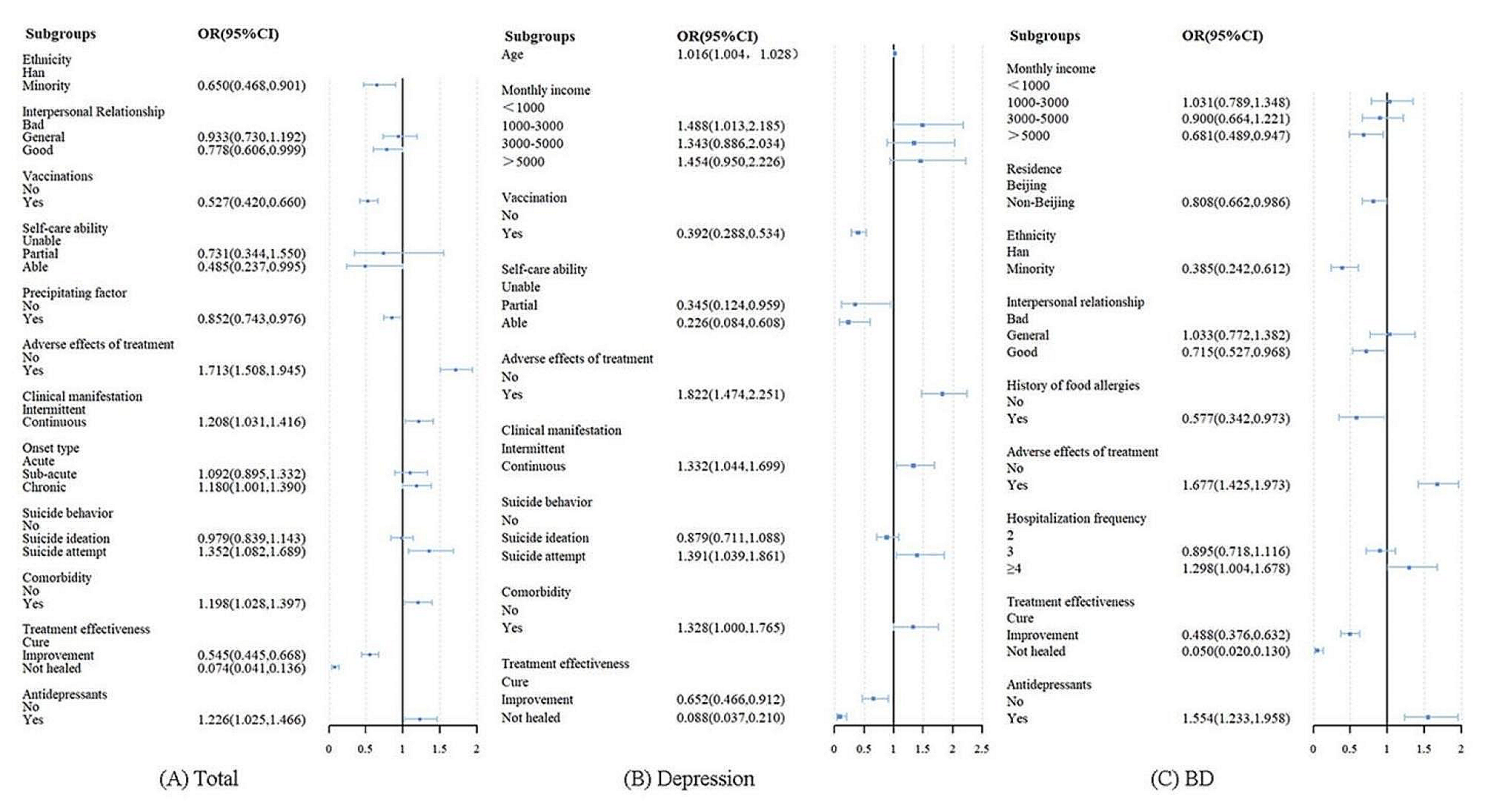
GEE model of LOS among repeatedly hospitalized patients with different disease types

Using all hospitalization data of repeatedly hospitalized patients with mood disorders, a GEE model was employed to incorporate sociodemographic characteristics, medical history, disease-related characteristics and treatment-related characteristics. The short LOS group was taken as the reference group in the GEE model. Figure 2A presents the results of the GEE model on the influencing factors of LOS among total patients. In terms of demographic variables, the study revealed that ethnic minority patients tended to have short LOS (OR = 0.650, 95%CI: 0.468, 0.901). And those with good interpersonal relationships (OR = 0.778, 95%CI: 0.606, 0.999) were obviously less likely to develop long LOS compared to patients with poor interpersonal relationships.

And in terms of medical history, history of vaccinations was strongly correlated with LOS. Patients with a history of vaccinations were more likely to have short LOS compared to unvaccinated patients, with an OR of 0.527 (95%CI: 0.420, 0.660). As for disease-related characteristics, patients who experienced adverse effects of treatment (OR = 1.713, 95%CI: 1.508, 1.945), those with continuous clinical characteristics (OR = 1.208, 95%CI: 1.031, 1.416), and those who had suicide attempt (OR = 1.352, 95%CI: 1.082, 1.689) showed a dramatically higher risk of long LOS compared to patients without adverse effects of treatment, presented intermittent clinical characteristics and never committed suicide. A negative correlation was found between disease precipitating factor (OR = 0.852, 95%CI: 0.743,0.976) and LOS. Additionally, comorbidity, treatment effectiveness and whether use antidepressants also affected the LOS among mood disorder patients. Patients who had comorbidity were more likely to stay longer in hospital (OR = 1.198, 95%CI: 1.028, 1.397).

Further analysis by different types of mood disorders indicated that there were discrepancies in the factors affecting LOS in patients with depression and patients with BD, as shown in Fig. 2B and C. Depressed patients who were elderly (OR = 1.016, 95%CI: 1.004,1.028), had low monthly income (OR = 1.488, 95%CI: 1.013,2.185), experienced adverse effects of treatment (OR = 1.822, 95%CI: 1.474,2.251), had continuous clinical characteristics (OR = 1.332, 95%CI: 1.044,1.699), attempted suicide (OR = 1.391, 95%CI: 1.039,1.861) and had comorbidity (OR = 1.328, 95%CI: 1.000,1.765) tended to have longer hospital stays. Conversely, depressed patients with a history of vaccinations (OR = 0.392, 95%CI: 0.288,0.534), and had the ability to care for themselves (OR = 0.226, 95%CI: 0.084,0.608) tended to have short LOS. As for treatment effectiveness, improvement (OR = 0.652, 95%CI: 0.466,0.912) and not healed (OR = 0.088, 95%CI: 0.037,0.210) was associated with short LOS among patients with depression. Slightly different from patients with depression, for BD patients, factors such as monthly income, residence, ethnicity, interpersonal relationship, food allergies history, vaccination history, adverse reaction in the treatment, hospitalization frequency, treatment effectiveness and whether use antidepressants were found to associated with LOS. BD patients who experienced adverse effects of treatment (OR = 1.677, 95%CI: 1.425,1.973), the number of repeated hospitalizations ≥ 4 (OR = 1.298, 95%CI: 1.004,1.678) and used antidepressants (OR = 1.554, 95%CI: 1.233,1.958) were more likely to stay longer in hospital.

**Fig. 3:**
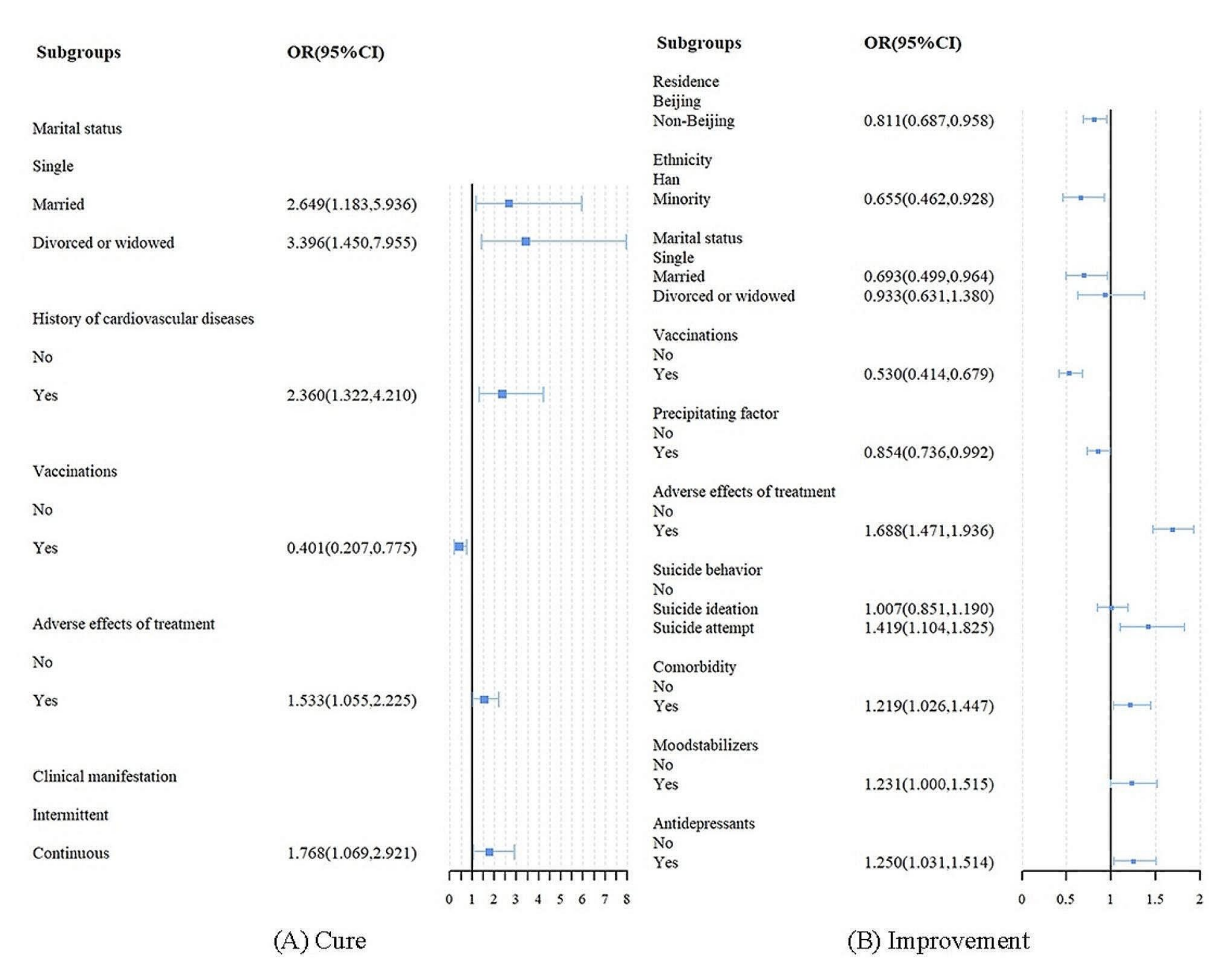
GEE model of LOS among patients with different treatment effectiveness

Stratified analyses were performed based on different treatment effectiveness at the initial hospitalisation, but patients who were not healed were excluded due to the limited sample size and lack of validity of the analyses. As Fig. 3A shows, cured patients who were married, divorced or widowed stayed longer than those who were single. The risk factors of long LOS also included history of cardiovascular diseases, adverse effects of treatment and continuous clinical manifestation. History of vaccination was negatively related to the long LOS. Among the improved patients who were minority, not resident in Beijing, married, had the history of vaccination and precipitating factor tended to be stay shorter in hospital. The adverse effects of treatment, suicide attempt, comorbidity, use of moodstabilizers and antidepressants were positively associated with long LOS in hospital.

## Discussion

### The LOS of study population

In this study, we retrospectively analyzed electronic medical records of repeatedly hospitalized patients with mood disorders in Beijing Anding Hospital to identify variables associated with LOS. Our research indicated that the mean LOS during the first hospitalization of patients with mood disorders was 32.12 days, which is shorter than the mean LOS among all mental disorder patients (36.1 days) previously reported in United Kingdom [8] and among patients with BD (37.0 days) reported in China [18], but slightly higher than the LOS (26.5 days) among adult patients with mood disorders reported in Canada [16]. The disagreement of LOS in hospital among studies may be explained by the difference in study population and the hospital. The severity of diseases was different in different regions and ethnic groups. Previous study also showed that for general hospitals, the proportion of short LOS is larger than long-term stay [19]. Besides, the bed capacity in hospital is also influencing the LOS of patients. A study conducted on the LOS in Ireland from 2010 to 2015 and found that a higher level of bed supply is associated with longer LOS [20].

### Sociodemographic characteristics

Age was found to be positively associated with LOS in patients with depression. However, this relationship was not found in patients with BD, which corroborates previous researches [11, 21]. In our study, the mean age of patients with BD and depression was 32.06 and 45.01 years, respectively. Depressed patients were significantly older than patients with BD. We hypothesized the association between LOS and age may be more pronounced in older age group. But a further study is needed to validate this conjecture. One possible explanation for the association between age and LOS is that family caregivers of older patients may be too busy to care for them at home, resulting in a reduction of family support and a longer LOS [22]. Additionally, older patients often have poor health and multiple comorbid conditions, which may contribute to longer LOS [23, 24].

Minority patients and with good interpersonal relationships were more likely to have shorter LOS. Conversely, Newman et al [25] conducted a study on factors associated with LOS in London and found that minority patients were associated with longer LOS. And a study demonstrated there was no relation between LOS and ethnicity [26]. The reason for the results may be due to differences in physical constitution and living habits among different ethnic groups. Patients with good interpersonal relationship have access to adequate social support, which had been shown in numerous studies to improve mental health and prevent relapse [27, 28]. This finding suggests that fostering positive social connectedness among patients with mental disorders should be a priority.

The study found that high income was associated with longer LOS in patients with BD, and low income was related to shorter LOS in patients with depression. T. Astell-Burt et al [29] analyzed the impact of income on psychological distress and found that individuals with lower incomes had greater odds of experiencing psychological distress, while those with higher incomes tended to have better mental health. On the other hand, patients with higher income may be more concerned about longer LOS leading to discrimination or even dismissal from their workplace [30].

### Medical history

The presence of cardiovascular diseases history was positively associated with an increased LOS among patients with BD. A study had shown that individuals with severe mental illness had a shorter life expectancy compared to the general population, with physical illnesses being the main contributor to this excess mortality [31]. People with BD are particularly susceptible to cardiovascular diseases [32], which further increases their risk of mortality. When BD patients also suffer from cardiovascular diseases, it can pose a challenge for the doctors to predict patient’s response to the treatment and medication [33], which may keep patients stay in the hospital longer for medical observation.

Our study revealed an interesting finding regarding the relationship between LOS and vaccination history among mood disorder patients. In our study, the vaccination referred to any of the vaccines in the National Immunization Program (NIP). Currently, there is a lack of research exploring the relationship in this specific population. Based on our hypothesis, patients who had received the vaccine demonstrated a higher level of openness to new things, and thus more cooperative with the treatment, potentially leading to a faster resolution of their condition and consequently shorter hospital stays. On the other hand, patients who had not been immunized might experience contraindications and poorer health, resulting in longer LOS.

### Disease-related characteristics

The study found that patients with precipitating factor had shorter LOS while those with continuous clinical manifestation had longer LOS. However, there is a lack of research on the relationship between precipitating factor, clinical manifestation, and LOS among mental disorder patients. Mood disorders can be triggered by various factors including stress, insomnia, and adverse experiences [34–37]. It is possible that identifying definite precipitating factor could aid in quicker diagnosis and treatment selection, ultimately reducing LOS.

Depression patients who are capable of taking care of themselves were more likely to have short LOS in our research. According to a study conducted on community patients in China, impaired self-care was found to be present in individuals with severe mental disorders [38]. We hypothesized that patients with a higher level of self-care ability may have milder symptoms and therefore tend to have a shorter LOS. However, it is important to note that our study did not establish a direct causal relationship.

Adverse effects of treatment and suicide attempt were related to long LOS in this research. Previous study indicated that adverse effects was one of the most reported factors associated with medication adherence [39]. Adverse effects such as drug adverse reaction result in poor adherence, which prolong the LOS. The result of suicide attempt was in contrast to a previous study [40]. This inconsistency might be caused by difference in the study population. In our study, repeatedly hospitalized patients with suicide attempt proved that patients had severe mental illness symptoms, leading to longer treatment time.

Agreement with previous studies, our research found that patients with comorbidity were more likely to have long LOS. A retrospective study was conducted in Portugal revealed medical comorbidity was associated with an average increase of 3.5 days in LOS compared to patients without medical comorbidity [41]. It has been suggested that the presence of physical medical problems places a greater burden on psychiatric patients, leading to poorer outcomes for their psychiatric condition, increased severity of symptoms, and higher incidence of non-compliance with treatment [24].

### Treatment-related characteristics

The effective of treatment had been hypothesised to predict LOS in hospital. In our study, improved and not healed patients stayed shorter in hospital compared with cured patients. We presume that the lack of effectiveness in treatment may influence patients’ confidence in the treatment, they are more likely to leave against medical advice. Additionally, patients who desired a more comprehensive treatment for their psychiatric symptoms tended to have longer hospital stays. This finding contrasts with a previous study that found a longer LOS in hospital for patients with a poor response to treatment [42]. Further research is needed to establish a clear causal relationship.

Consistent with the published researches [10, 18], among BD patients who used antidepressants were more likely to stay longer in hospital. In the duration of BD, depressive state is a painful phase in the most patients. Seriously, depressive states tend to induce further symptoms, including irritations, insomnia, and various somatic complaints [43]. For this reason, a longer treatment process is necessary to address the depressive state of patients with BD.

There were some similarities in the relationship between LOS and characteristics across the two effectiveness of treatment groups, but there were some noticeable differenceswhich should be interpreted with caution. Our findings provided information to the psychiatrists that they could give timely and early attention to specific factors that sustain long-term hospitalization when dealing with different types of hospitalized patients with mood disorders.

## Conclusion

In conclusion, the LOS in repeatedly hospitalized patients with mood disorders was affected by a combination of factors including sociodemographic characteristics, disease-related characteristics, medical history and treatment-related characteristics. It is noteworthy that there are discrepancies in the factors affecting LOS in patients with BD and those with depression, and disease-specific factors should be considered when evaluating LOS in patients with different types of mood disorders. Identifying factors that contribute to prolong hospital stays is a critical step in addressing resource constraints in mental health care and can provide valuable clues for optimizing management of the psychiatry department.

## Limitations

This study had several limitations that need to be addressed. Firstly, our study was a single-center study, restricting the ability to make comprehensive generalizability of the results to other regions and populations in China. In the future study, expanding the sample size and including hospitals in more regions can enhance generalizability. Secondly, due to this study was conducted only for the retrospective analysis of electronic medical records, it was not possible to establish explicit causality between LOS and relevant factors. Thirdly, the absence of biochemical variables, imaging findings and educational level of patients in the database might hinder the exploration of other potential factors of LOS. Future research can work on the relationship between these aspects and LOS. Finally, it is difficult to assess the Charlson Comorbidity Index in this study because our data is obtained from EMRs, which do not directly record information about Charlson Comorbidity Index. However, we incorporated the presence or absence of comorbidities into the scope of our research, which partially helps to explain the relationship between comorbidities and LOS in hospital to some extent.

## Data Availability

The data that support the findings of this study are not publicly available due to privacy or ethical restrictions.

## Abbreviations

BD: Bipolar disorder
EMRs: Electronic medical records
GEE: Generalized estimating equation
ICD-10: International Classification of Diseases-10th Revision
LOS: Length of stay
MECT: Modified electroconvulsive therapy
NIC: National Immunization Program
